# Mechanical energy on anaerobic capacity during a supramaximal treadmill running in men: Is there influence between runners and active individuals?

**DOI:** 10.14814/phy2.15564

**Published:** 2023-03-10

**Authors:** Alessandro Moura Zagatto, Joel Abraham Martínez González, Rodrigo Araujo Bonetti de Poli, Fabio Augusto Barbieri, Leonardo de los Santos Bloedow, Leonardo Peyré‐Tartaruga

**Affiliations:** ^1^ Post‐Graduate Program in Movement Sciences, Department of Physical Education, School of Sciences Sao Paulo State University (UNESP) Bauru SP Brazil; ^2^ Universidad Autónoma del Estado de México Toluca Mexico; ^3^ Universidade Federal do Rio Grande do Sul, Exercise Research Laboratory Porto Alegre RS Brazil

**Keywords:** blood lactate, excess postexercise oxygen consumption, mechanical work, non‐mitochondrial metabolic pathways, physical fitness level, time to effort failure

## Abstract

This study verified whether mechanical variables influence the anaerobic capacity outcome on treadmill running and whether these likely influences were dependent of running experience. Seventeen physical active and 18 amateur runners, males, performed a graded exercise test and constant load exhaustive running efforts at 115% of intensity associated to maximal oxygen consumption. During the constant load were determined the metabolic responses (i.e., gas exchange and blood lactate) to estimate the energetic contribution and anaerobic capacity as well as kinematic responses. The runners showed higher anaerobic capacity (16.6%; *p* = 0.005), but lesser time to exercise failure (−18.8%; *p* = 0.03) than active subjects. In addition, the stride length (21.4%; *p* = 0.00001), contact phase duration (−11.3%; *p* = 0.005), and vertical work (−29.9%; *p* = 0.015). For actives, the anaerobic capacity did not correlate significantly with any physiologic, kinematic, and mechanical variables and no regression model was fitted using the stepwise multiple regression, while to runners the anaerobic capacity was significantly correlated with phosphagen energetic contribution (*r* = 0.47; *p* = 0.047), external power (*r* = −0.51; *p* = 0.031), total work (*r* = −0.54; *p* = 0.020), external work (*r* = −0.62; *p* = 0.006), vertical work (*r* = −0.63; *p* = 0.008), and horizontal work (*r* = −0.61; *p* = 0.008), and the vertical work and phosphagen energetic contribution presented a coefficient of determination of 62% (*p* = 0.001). Based on findings, it is possible to assume that for active subjects, the mechanical variables have no influence over the anaerobic capacity, however, for experienced runners, the vertical work and phosphagen energetic contribution have relevant effect over anaerobic capacity output.

## Abstract 2

Investigate whether mechanical variables influence the anaerobic capacity outcome on treadmill running and whether these likely influences depended on running experience. Kinematic variables are not correlated with anaerobic capacity for untrained, but for trained, there are negative correlation with external power, and total, external, vertical, and horizontal works and the only vertical work and phosphagen energy parameters could be used to prediction of anaerobic capacity.

## 1 INTRODUCTION

The anaerobic capacity is defined as the maximal amount of energy which can be resynthesized by non‐mitochondrial metabolic pathways (i.e., glycolytic and phosphagen pathways) in a specific effort performed until failure (Zagatto, Bertuzzi, et al., 2016; Zagatto, Leite, et al., 2016). Considering the non‐mitochondrial metabolic pathways have actions predominant in the first seconds of an effort acting until approximately 45 s for a maximal effort (Hargreaves & Spriet, 2020; Zagatto et al., 2022), the anaerobic capacity has been significantly correlated with the performance recorded in short‐term events (Nevill et al., 2008; Ramsbottom et al., 1994, 2001; Zagatto, Miyagi, et al., 2017; Zagatto, Nakamura, et al., 2017).

Classically, the anaerobic capacity has been estimated by maximal accumulate oxygen deficit (Medbo et al., 1988; Miyagi et al., 2017; Noordhof et al., 2010; Zagatto et al., 2011; Zagatto & Gobatto, 2012; Zagatto, Bertuzzi, et al., 2016; Zagatto, Leite, et al., 2016). However, in the last decade, some studies (Bertuzzi et al., 2010; de Poli et al., 2019; Miyagi et al., 2017, 2018; Zagatto, Bertuzzi, et al., 2016; Zagatto, Leite, et al., 2016; Zagatto et al., 2019) have used the classical findings reported by Margaria et al. (1933) and by Di Prampero and Ferretti (1999) to estimate the energetic contribution (i.e., expressed in oxygen equivalent) from phosphagen pathway by the fast component of excess postexercise oxygen consumption and the net lactate accumulated to estimate the glycolytic energetic contribution, therefore assuming the anaerobic capacity as the sum of oxygen equivalent from both the energetic pathways (Bertuzzi et al., 2010; de Poli et al., 2020; Miyagi et al., 2017; Zagatto et al., 2010, 2019; Zagatto, Bertuzzi, et al., 2016; Zagatto, Leite, et al., 2016; Zagatto, Miyagi, et al., 2017; Zagatto, Nakamura, et al., 2017).

Nevertheless, since 1990s and till now is usual to find studies using mechanical performance variables to estimate the anaerobic fitness, such repeated jumps protocols (Bosco et al., 1983), outcomes from Wingate cycling test (Popadic Gacesa et al., 2009; Zagatto et al., 2008, 2009), the running anaerobic sprint test (i.e., adaptation of Wingate test for running) (Andrade et al., 2015; Brocherie et al., 2015; Milioni et al., 2017; Zagatto et al., 2009), and others. However, Minahan et al. (2007) reported that higher anaerobic power from Wingate test does not indicate a greater anaerobic capacity estimated by maximal accumulate oxygen deficit. Likewise, Andrade et al. (2015) showed that the parameters from running anaerobic sprint test did not have association with maximal accumulate oxygen deficit, suggesting that estimates of mechanical power performance should not be used to estimate the anaerobic capacity.

One possible reason for the disagreement between the methods for estimating anaerobic capacity from measures of mechanical power could be due to amount of energy expended from the muscle oxidative metabolism. Beneke et al. (2002) reported that during Wingate test, the fraction of the energy from oxidative metabolism was approximately 18%, while Milioni et al. (2017) demonstrated that during running anaerobic sprint test, the oxidative fraction corresponded to 38% when assuming the whole test including the resting intervals and 20% when assumed only the efforts time (i.e., no considering the resting intervals). Therefore, the testing to estimate the anaerobic capacity using mechanical power outcomes are limited because of different energetic demand from oxidative pathway engaged in different exercises settings (i.e., duration, intensity, and effort mode).

The analysis of mechanical and metabolic power at high‐intensity efforts have been extensively used in the literature in order to evaluate and prescribe training for athletes as well as to understand the mechanisms underlying the athletic performance (Bosco et al., 1983; de Poli et al., 2020; Margaria et al., 1966; Morin et al., 2012; Popadic Gacesa et al., 2009; Zagatto et al., 2009). Although the relationships between mechanical and metabolic power measurements are well established under endurance conditions (Cavagna & Kaneko, 1977; Lacour & Bourdin, 2015), there are still many questions during high‐intensity exercise. This scarcity is partly because of non‐stable and transient conditions under which exercise is performed, making the mechanical (Peyré‐Tartaruga et al., 2021) and metabolic (Zamparo et al., 2019) measurements challenging.

In running at high‐intensity conditions, horizontal external power is higher than vertical external power (Cavagna & Kaneko, 1977) probably associating with anaerobic capacity. In these high‐speed conditions, intrasubject adjustments of speed seem to be more related to changes in stride frequency than stride length (Cavagna & Kaneko, 1977). In turn, stride frequency seems to be related to internal mechanical power (Minetti, 1998) and horizontal external power (Cavagna et al., 2008), and stride length is related to external mechanical work (Cavagna et al., 2008). Furthermore, mechanical adjustments are observed in order to mitigate deterioration in running energy expenditure at high‐metabolic running intensities. Trained triathletes reduce stride length and increase stride frequency during the onset of running fatigue so that mechanical efficiency is maintained (da Rosa, de Oliveira, et al., 2019). Likewise, the elastic running mechanism that directly affects mechanical efficiency is improved in high performing 3000 m runners compared to low performing runners (da Rosa, Oliveira, et al., 2019). However, the specific responses of running‐specific training compared to trained athletes of other disciplines are not yet known, especially at supramaximal metabolic intensities.

Therefore, this study verified whether mechanical variables influence the anaerobic capacity outcome on treadmill running and whether these likely influences depended on running experience. To investigate this purpose, we have applied the stepwise linear regression model using the anaerobic capacity as the dependent variable and the physiologic, kinetics, and kinematic variables as independent variables to attempt to fit a predictive model. In addition, we also aimed to verify the relevance of these variables on short‐term running performance. We hypothesized that the vertical oscillation and contact time should be lower in trained runners than non‐runner athletes due to a more adapted running technique on supramaximal conditions in runners.

## 2 METHODS

### 2.1 Participants

The required sample size was estimated using G*power software. The input parameters used for *t*‐test family were as follows: alpha = 0.05 and power = 0.90. Using the findings from Zagatto, Miyagi, et al. (2017); (Zagatto, Nakamura, et al., 2017) that reported an effect size of 1.17 for the anaerobic capacity measured on treadmill running between active (3.45 ± 0.43 L O_2_) and endurance trained (4.11 ± 0.67 L O_2_) subjects, the minimum required sample size calculated was of 17 participants for each independent group.

Seventeen physically active (21.9 ± 3.0 years; height of 174.0 ± 4.7 cm, body mass of 72.5 ± 9.4, and maximal oxygen consumption of 42.8 ± 3.3 ml·kg^−1^·min^−1^) and 18 amateur runners (34.1 ± 3.7 years; height of 178.6 ± 4.9 cm, body mass of 72.0 ± 5.6, and maximal oxygen consumption of 55.9 ± 5.5 ml·kg^−1^·min^−1^), healthy male volunteers, with absence of vascular and metabolic disorders, musculoskeletal and joint injuries, and recent (<6 months) and regular use of nutritional ergogenic supplements were recruited to participate in this study voluntarily.

The active group was composed by untrained participants, who performed sporadic physical activities such as soccer, running, and cycling. In addition to the abovementioned criteria, each runner should have at least 2 years of training experience and be training at the moment of the study data collection. Before any test, we informed the volunteers about the procedures and risks, and they signed an informed consent document before beginning the experimental procedures. All the procedures in this study were approved by the Ethics Research Committee of São Paulo State University (Process Number: 1.846.716), and the study was conducted according to the Declaration of Helsinki.

During the procedures, the volunteers were instructed to avoid nutritional performance‐enhancing aids such as caffeine, sodium bicarbonate, and others. They also were informed to avoid performing strenuous exercise 24 h before each exercise session. Further, the volunteers reported that they had not taken ergogenic substances like chronic creatine or beta‐alanine ingestion in the previous 3 months. Finally, to eliminate any influence of circadian variation on mechanical and physiologic outcomes, each subject completed all trials at the same time of the day in controlled environmental conditions regarding temperature (21.4 ± 0.7°C) and relative humidity (44.3 ± 5.6%).

### 2.2 Experimental design

This study was composed of four visits to the laboratory, separated by 48 h. On the first visit, the participants performed a graded exercise test to measure the maximal oxygen consumption (V̇O_2max_) and the velocity associated with V̇O_2max_ (vV̇O_2max_), followed by a V̇O_2max_ verification test. In the subsequent three visits, the participants underwent constant load supramaximal exhaustive running effort at 115% of vV̇O_2max_, which was performed until effort failure (Zagatto, Bertuzzi, et al., 2016; Zagatto, Leite, et al., 2016; Zagatto, Miyagi, et al., 2017; Zagatto, Nakamura, et al., 2017). The first two trials were conducted as familiarizations to the tests. In the third trial, we collected the time to effort failure (TTE), the metabolic variables, and kinematics during the effort. Before each test, a standardized warm up was performed lasting 5 min at 8 km·h^−1^. All the efforts were performed on a motorized treadmill (ATL, Inbramed) 1% slope (Jones & Doust, 1996). They were verbally encouraged and wore a safety belt attached to their chest to ensure maximal effort.

### 2.3 Physiologic measurements

The gas exchange responses were measured breath‐by‐breath using a stationary gas analyzer (Quark CPET, Cosmed), coupled with a heart rate transmitter (Wireless HR 138 Monitor, Cosmed). The gas analyzer was calibrated before each test using an ambient air sample and a high‐precision gas mixture (3.98% CO_2_, 16.02% O_2_, and balanced N_2_; White Martins Gases Industriais Ltda), whereas the pneumotachograph was calibrated through a 3 L syringe (Hans Rudolf), in accordance with the manufacturer's instructions. For analysis of cardiorespiratory responses, the raw data were smoothed using five points moving average and were interpolated every 1 s using the software OriginPro 8.0.

Furthermore, to determine the blood lactate concentration ([La^−^]), blood samples from the earlobe (25 μl) were taken in the 3rd and 5th min after the graded exercise test and at rest, 3rd, 5th, and 7th min after constant‐load supramaximal effort, transferred to Eppendorf tubes containing 50 μl of 1% sodium fluoride, and kept frozen at −20°C until the assay. The samples were analyzed in duplicate using a biochemical analyzer YSI 2900 (Yellow Spring Instruments, EUA) with a measurement error ranging from ±2%. We used the Borg scale (6–20) to assess the rating of perceived exertion (RPE) on every effort.

### 2.4 Graded exercise test

The graded exercise test started at 8 km·h^−1^ and it was incremented in 1.5 km·h^−1^ every 2 min (Zagatto, Bertuzzi, et al., 2016; Zagatto, Leite, et al., 2016). Additionally, a verification test for V̇O_2max_ was performed 5 min after the end of graded exercise test (passive recovery), at 110% of vV̇O_2max_ until the effort failure. The gas exchange responses were measured during the whole test. The highest 30 s‐averaged oxygen uptake (V̇O_2_) attained in graded exercise test was assumed as V̇O_2max_, considering the verification of plateau as the main criterion (changes in V̇O_2_ < 2.1 ml·kg^−1^·min^−1^ between the last and penultimate exercise stages). As a secondary criterion to determine V̇O_2max_, we followed a traditional one: (i) maximal heart rate > age‐predicted maximum (220 age), (ii) maximal [La^−^] > 8 mmol·L^−1^, and (iii) respiratory exchange ratio >1.10. The highest value of the last 30 s of verification testing was calculated and compared with the highest V̇O_2_ value obtained in the graded exercise text.

The vV̇O_2max_ was assumed as the minimal exercise intensity in which V̇O_2max_ was attained. In case of effort failure without to complete an exercise stage, the vV̇O_2max_ was assumed according to recommendation of Kuipers et al. (1985).

### 2.5 Constant‐load supramaximal effort and anaerobic capacity assessment

Prior to each constant‐load supramaximal trial, the participants remained at rest seated in a chair for 10 min in order to determine the resting values of V̇O_2_ (assuming the V̇O_2_ average of the last 2 min of rest) and the [La^−^]. After a warm up (abovementioned), the individuals performed the constant‐load supramaximal effort at 115% of vV̇O_2max_ until the task failure, which was assumed as the incapacity to keep the running intensity (Zagatto, Bertuzzi, et al., 2016; Zagatto, Leite, et al., 2016; Zagatto, Miyagi, et al., 2017; Zagatto, Nakamura, et al., 2017; Zagatto et al., 2022). The TTE was recorded. The gas exchange responses were also measured during the effort and 10 min after the end of the test to estimate the fast component of excess postexercise oxygen consumption. In addition, blood samples were withdrawn in rest and after exercise to determine the [La^−^] (i.e., for details, see above).

The V̇O_2_ measured after exercise was adjusted by a biexponential function (Equation 1) using the OriginPro 2017 software (Origin Lab Corporation, Microcal). The oxygen equivalent corresponding to phosphagen energy pathway (*E*_PCr_) was estimated by fast component of excess postexercise oxygen consumption, which was calculated by multiplication of V̇O_2_ amplitude 1 and time constant 1 from biexponential adjustment (Brisola et al., 2015; Margaria et al., 1933; Miyagi et al., 2017; Zagatto, Bertuzzi, et al., 2016; Zagatto, Leite, et al., 2016; Zagatto, Miyagi, et al., 2017; Zagatto, Nakamura, et al., 2017; Zagatto et al., 2022). The oxygen equivalent from glycolytic energy pathway (*E*_[La_^−^_]_) was estimated by net lactate concentration accumulation (i.e., difference between peak [La^−^] and the baseline [La^−^]; ∆[La^−^]), assuming an oxygen equivalent 3.0 ml·kg^−1^ for each ∆[La^−^] of 1 mmol·L^−1^ (Brisola et al., 2015; Di Prampero & Ferretti, 1999; Miyagi et al., 2017; Zagatto, Bertuzzi, et al., 2016; Zagatto, Leite, et al., 2016; Zagatto, Miyagi, et al., 2017; Zagatto, Nakamura, et al., 2017; Zagatto et al., 2022). Therefore, the anaerobic capacity was assumed as the sum of *E*_PCr_ and *E*_[La_^−^_]_ (Brisola et al., 2015; Milioni et al., 2016, 2017; Miyagi et al., 2017; Zagatto & Gobatto, 2012; Zagatto, Bertuzzi, et al., 2016; Zagatto, Leite, et al., 2016; Zagatto, Miyagi, et al., 2017; Zagatto, Nakamura, et al., 2017; Zagatto et al., 2022). The oxygen equivalent from oxidative metabolism (*E*_OXID_) also was estimated considering the V̇O_2_ area under the curve by trapezoidal method (Brisola et al., 2015; Milioni et al., 2017; Miyagi et al., 2017; Zagatto, Bertuzzi, et al., 2016; Zagatto, Leite, et al., 2016; Zagatto, Miyagi, et al., 2017; Zagatto, Nakamura, et al., 2017; Zagatto et al., 2022).

(1) $$\dot{V}O_{2\left(t\right)}=\text{baseline}\;\dot{V}O_{2}+A_{1}\left[e^{-\left(t/\delta \right)/\tau 1}\right]+A_{2}\left[e^{-\left(t/\delta \right)/\tau 2}\right],$$

where $\dot{V}O_{2\left(t\right)}$ is the oxygen uptake at time *t*, *A* is the V̇O_2_ amplitude, δ is the time delay, and *τ* is the time constant. 1 and 2 represent the fast and slow components, respectively, and the fast component of excess postexercise oxygen consumption was calculated by multiplying amplitude 1 and time constant 1.

### 2.6 Kinematic measurement during supramaximal effort

#### 2.6.1 Acquisition and processing of kinematic data

The kinematic measurement was recorded during constant‐load supramaximal effort at 115% of vV̇O_2max_ until the task failure. Therefore, 39 reflective markers were placed on each subject in accordance with the Plug‐in‐Gait Full Body (Vicon®) model. The kinematic data were continuously acquired during whole trial using eight cameras Vicon System (Bonita System Cameras, Vicon Motion System®, MX System, EUA) and the motion capture was collected at a sampling rate of 250 Hz.

#### 2.6.2 Data analyses

The spatial coordinates of the reflective markers were reconstructed in the Nexus program (Vicon Oxford) as position data establishing 11 segments through which it was possible to determine the body center of mass and segment positions during the entire treadmill supramaximal effort (Winter, 1979).

The data were filtered using a 2° order low‐pass Butterworth filter with a cutoff frequency of 5 Hz and analyzed using a custom algorithm developed in MatLab (2012b, Mathworks Inc.).

#### 2.6.3 Calculation of the mechanical variables

Ten consecutive strides were selected to the following analysis and the mechanical and spatiotemporal values were averaged. The mechanical work was calculated according to Peyré‐Tartaruga et al. (2021). From the three‐dimensional positions of the 39 reflective markers, we built a spatial model of 11 rigid segments. The characteristics of each body segment (center of mass position and radius of gyration) were determined according to De Leva (1996). The 3D trajectory of the body center of mass was calculated through the spatial coordinates of reflective markers. Based on the body center of mass data, the time course of potential (PE) and kinetic (EK) energies were computed to obtain the total mechanical energy (ME), as follows (Equation 2).

(2) $$\mathrm{ME}=\mathrm{PE}+{\mathrm{EK}}_{X}+{\mathrm{EK}}_{Y}+{\mathrm{EK}}_{Z},$$

where *X* is the antero‐posterior axis, *Y* the mediolateral axis, and *Z* the vertical axis (Cavagna & Kaneko, 1977).

The work necessary to lift and accelerate the body center of mass within the environment, the external work, was calculated as the summation of all positive increases in the time course of the ME. We also determined the work necessary to lift (vertical work = EP + EK_*Z*_) and accelerate (horizontal work = EK_*X*_) the body center of mass. The work necessary to rotate and accelerate the limbs with respect to body center of mass, the internal work, was calculated through the summation of all increases in rotational energy from the body segments and all increases in translational energy from the body segments relative to the body center of mass (Cavagna & Kaneko, 1977). In addition, it was assumed the transfer of energy between leg and thigh segments, as well as between arm and forearm as defined previously (Willems et al., 1995). Finally, for the calculation of the total work which is characterized by the work required to sustain locomotion, it was obtained by the sum of the external and internal work (Cavagna et al., 1997). The total, external, internal power, and their respective components were obtained through the rate calculated from total, external, internal work, and the time of stride duration. The stride length was calculated as the distance between the first contact of the foot with the floor of the marker positioned on the heel and the first contact of the same made in the subsequent stride. The contact time was determined by the height of the heel's reflective marker at the time the foot meets the treadmill until the moment the same marker reaches a known height where the foot has its last contact with the treadmill. The aerial phase duration was determined by the time between the moment that the 5° metatarsal's reflective marker leave the treadmill until the next contact of the heel's reflective marker of the same foot made in subsequent stride. The stride frequency was determined as the number of strides per second.

### 2.7 Statistical analysis

The results are presented as means ± standard deviations. For comparison between active and runner groups, unpaired *t*‐test was used and the difference between groups were also reported in percentage of changes (∆%) and Cohen's d effect size (ES). In order to find the best predictors, we first calculated the variance explained by the combination of biomechanical variables and the dependent variable (i.e., anaerobic capacity and TTE) with a stepwise multiple linear regression. In this case, the analysis was performed separately for each group and including all the biomechanical variables as predictors of the dependent variable. Then, we calculated a bivariate Pearson's correlations with data of each group separately. The correlation coefficient was classified as very weak to negligible (0 to 0.2), weak (0.2 to 0.4), moderate (0.4 to 0.7), strong (0.7 to 0.9), and very strong (0.9 to 1.0).

We calculated ESs as proposed by Hopkins with ES < 0.2 considered trivial, 0.2–0.5 small, 0.6–1.1 moderate, 1.2–1.9 large, and >2 very large. In this study, we assumed a significance level of 5%.

## 3 RESULTS

### 3.1 Physiologic and metabolic variables

The V̇O_2max_ (55.9 ± 5.5 ml·kg^−1^·min^−1^ for runners and 42.8 ± 3.3 ml·kg^−1^·min^−1^ for active subjects; ES = 2.2; ∆% = +30.6%) and vV̇O_2max_ (17.7 ± 1.1 km·h^−1^ for runners and 14.3 ± 1.1 km·h^−1^ for active subjects; ES = 1.82; ∆% = +27.8%) measured during graded exercise tests were significantly different between groups (*p* = 0.000000001 and *p* = 0.000000003, respectively). Based on the vV̇O_2max_ measurement, the exercise intensity at 115% of vV̇O_2max_ corresponded to 20.4 ± 1.4 km·h^−1^ for runners and 16.5 ± 1.3 km·h^−1^ for active groups.

**FIGURE 1:**
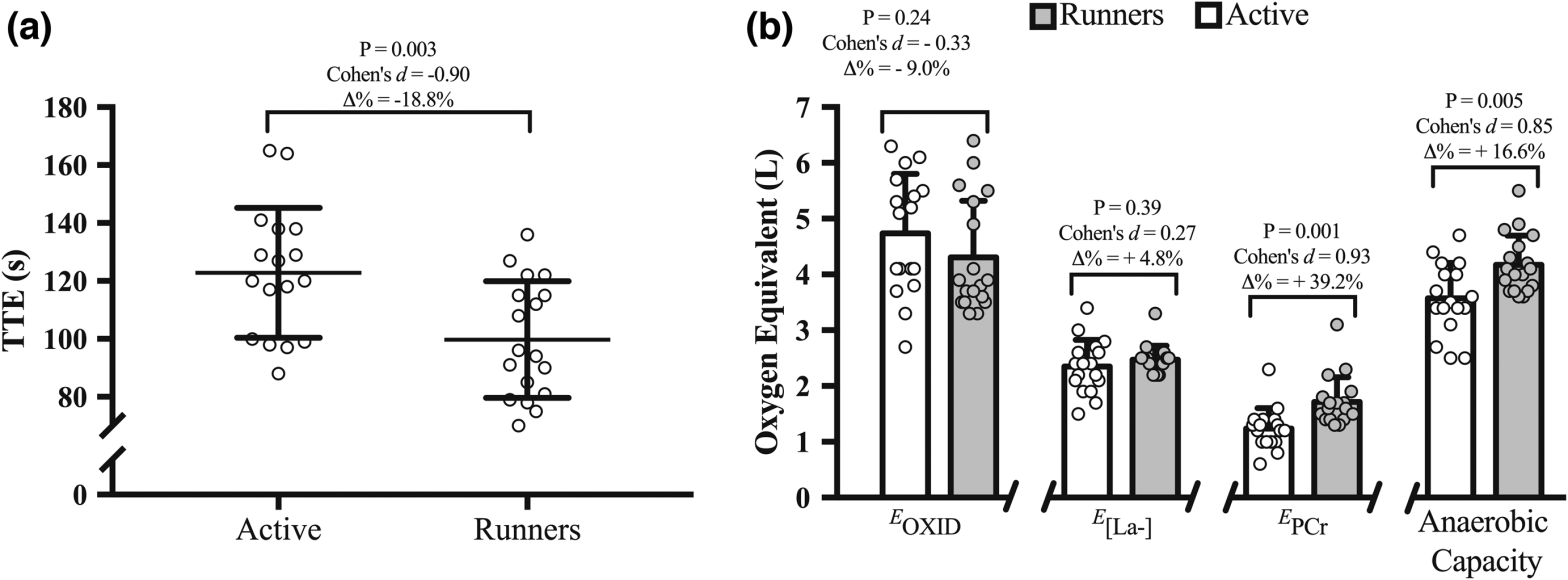
The (a) shows the mean and standard deviation of time to effort failure (TTE) recorded in the constant‐load supramaximal effort at 115% of vV̇O_2max_ and the (b) shows the oxygen equivalents estimated from the oxidative (*E*_OXID_), glycolytic (*E*_[La_^−^_]_), and phosphagen (*E*_PCr_) metabolisms as well as the anaerobic capacity. In both panels, each symbol represents one participant.

For exercise performed in the constant‐load supramaximal effort at 115% of vV̇O_2max_, the TTE was significantly lower (*p* = 0.03) for runners compared to active groups (Figure 1a), while the peak V̇O_2_ value was statistically higher (50.4 ± 5.6 ml·kg^−1^·min^−1^ vs. 41.8 ± 4.9 ml·kg^−1^·min^−1^, *p* = 0.00003), respectively. However, no statistical difference (*p* ≥ 0.245) was found for the RPE (18 ± 1 a.u. and 19 ± 1 a.u., respectively; ES = −0.54; ∆% = 3.5%) and resting [La^−^] (0.88 ± 0.22 mmol·L^−1^ and 0.82 ± 0.32 mmol·L^−1^, respectively; ES = 0.19; ∆% = +7.4%), peak [La^−^] (12.34 ± 1.39 mmol·L^−1^ and 11.67 ± 1.97 mmol·L^−1^, respectively; ES = 0.35; ∆% = +5.8%) and for the net [La^−^] accumulation (11.46 ± 1.35 mmol·L^−1^ and 10.84 ± 2.00 mmol·L^−1^, respectively; ES = 0.32; ∆% = +5.7%).

The Figure 1b shows the oxygen equivalents from the oxidative (*E*_OXID_), glycolytic (*E*_[La_^−^_]_), and phosphagen (*E*_PCr_) metabolisms, and it was found only greater value of *E*_PCr_ for runners' group (*p* = 0.001; i.e., higher A1 20.4 ± 3.7 ml and 15.9 ± 2.8 ml, respectively). In this way, the anaerobic capacity was 16.6% higher in runner group compared to active group (Figure 1b; *p* = 0.005).

### 3.2 Kinematic measurements

Table 1 shows the spatiotemporal parameters, and the runner group presented higher stride length (*p* = 0.00001) and shorter contact phase duration (*p* = 0.005), but no difference was found for stride frequency (*p* = 0.18) and the aerial phase duration (*p* = 0.95).

**TABLE 1:** Mean, standard deviation, and IC 95% of kinematic variables estimated during the constant‐load supramaximal effort

Variables	Active group (*N* = 17)	Runner group (*N* = 18)	ES	∆%
Spatiotemporal parameters				
Stride frequency (Hz)	1.54 ± 0.44	1.61 ± 0.15	0.36	3.9
Stride length (m)	2.93 ± 0.32	3.55 ± 0.39***	1.40	21.4
Aerial phase duration (s)	0.44 ± 0.04	0.45 ± 0.05	0.02	0.2
Contact phase duration (s)	0.21 ± 0.02	0.18 ± 0.03**	−0.76	−11.3

** *p* ≤ 0.01

*** *p* ≤ 0.001.

Table 2 shows the mechanical work and power variables calculated during constant‐load supramaximal effort. The runner group showed a lower vertical work (*p* = 0.015), but no other significant difference was found between groups for mechanical variables.

**TABLE 2:** Mean, standard deviation, and IC 95% of mechanical work and power variables estimated during the constant‐load supramaximal effort at 115% of vV̇O_2max_.

Variables	Active group (*N* = 17)	Runner group (*N* = 18)	ES	∆%
Work				
Total work (J·kg^−1^·m^−1^)	6.17 ± 2.25	6.49 ± 1.84	0.13	5.2
External work (J·kg^−1^·m^−1^)	1.85 ± 0.67	1.66 ± 0.82	−0.20	−10.4
Internal work (J·kg^−1^·m^−1^)	4.32 ± 1.78	4.83 ± 1.11	0.31	12.0
Vertical work (J·kg^−1^·m^−1^)	0.60 ± 0.27	0.42 ± 0.10**	−0.81	−29.9
Horizontal work (J·kg^−1^·m^−1^)	1.49 ± 0.79	1.31 ± 0.84	−0.18	−11.9
Trunk internal work (J·kg^−1^·m^−1^)	0.90 ± 0.47	0.84 ± 0.61	−0.09	−6.9
Upper internal work (J·kg^−1^·m^−1^)	0.24 ± 0.15	0.26 ± 0.17	0.10	8.5
Lower internal work (J·kg^−1^·m^−1^)	3.18 ± 1.38	3.74 ± 1.11	0.38	17.5
Power				
Total power (W·kg^−1^)	9.50 ± 3.55	10.10 ± 2.42	0.17	6.3
External power (W·kg^−1^)	2.85 ± 1.07	2.57 ± 1.09	−0.21	−9.8
Internal power (W·kg^−1^)	6.65 ± 2.77	7.53 ± 1.56	0.35	13.2

** *p* ≤0.01.

### 3.3 Pearson correlations

For runners group, the TTE was significant correlated with body mass (*r* = 0.50; *p* = 0.033), V̇O_2max_ (*r* = −0.60; *p* = 0.008), vV̇O_2max_ (*r* = − 0.62; *p* = 0.006), and peak [La^−^] (*r* = − 0.52; *p* = 0.026), while the anaerobic capacity was significant correlated with *E*_PCr_ (*r* = 0.47; *p* = 0.047), external power (*r* = − 0.51; *p* = 0.031), total work (*r* = − 0.54; *p* = 0.020), external work (*r* = − 0.62; *p* = 0.006), vertical work (*r* = − 0.63; *p* = 0.008), and horizontal work (*r* = −0.61; *p* = 0.008).

For active group, TTE was only correlated with *E*_OXID_ (*r* = 0.50; *p* = 0.042), while the anaerobic capacity did not correlate significantly with any physiologic, kinematic. and mechanical variables.

### 3.4 Stepwise multiple regression

We have used a stepwise multiple regression to investigate if and whom physiologic, kinematic, and mechanical variables are able to predict the TTE and mainly the anaerobic capacity in each subject group.

For the active group, the analyses showed a significant regression model for TTE (*F*_(5,11)_ = 45.79, *p* < 0.0001, *R* ^2^ = 0.95), but no variables were entered into the equation to predict the anaerobic capacity.

The *E*_OXID_ (*β* = 0.627; *t* = 7.94; *p* < 0.001), body mass (*β* = −1.009; *t* = −9.458; *p* < 0001), body height (*β* = 0.427; *t* = 4.205; *p* = 0.001), *E*_[La_^−^_]_ (*β* = 0.697; *t* = 8.617; *p* < 0.001), and contact phase duration (*β* = 0.248; *t* = 3.125; *p* = 0.10) were predictors of the TTE. No variables were entered into the equation for capacity anaerobic; therefore, no predictor model was fitted.

For the runner group, the analysis showed significant regression models for TTE (*F*_(2,15)_ = 8.892, *p* = 0.001, *R* ^2^ = 0.62) and for capacity anaerobic (*F*_(2,15)_ = 12.173; *p* = 0.001; *R* ^2^ = 0.62). For predicting the TTE, the constant was 4459.67 and variables entered into the model were the supramaximal intensity (*β* = −0.803; *t* = −4.656, *p* < 0.001) and age (*β* = −0.514; *t* = −2.982; *p* = 0.009). For predicting the anaerobic capacity, the constant was 47.327 and variables entered into the model were vertical work (*β* = −0.628; *t* = −3.936; *p* = 0.001) and *E*_PCr_ (*β* = 0.476; *t* = 2.988; *p* = 0.009).

**FIGURE 2:**
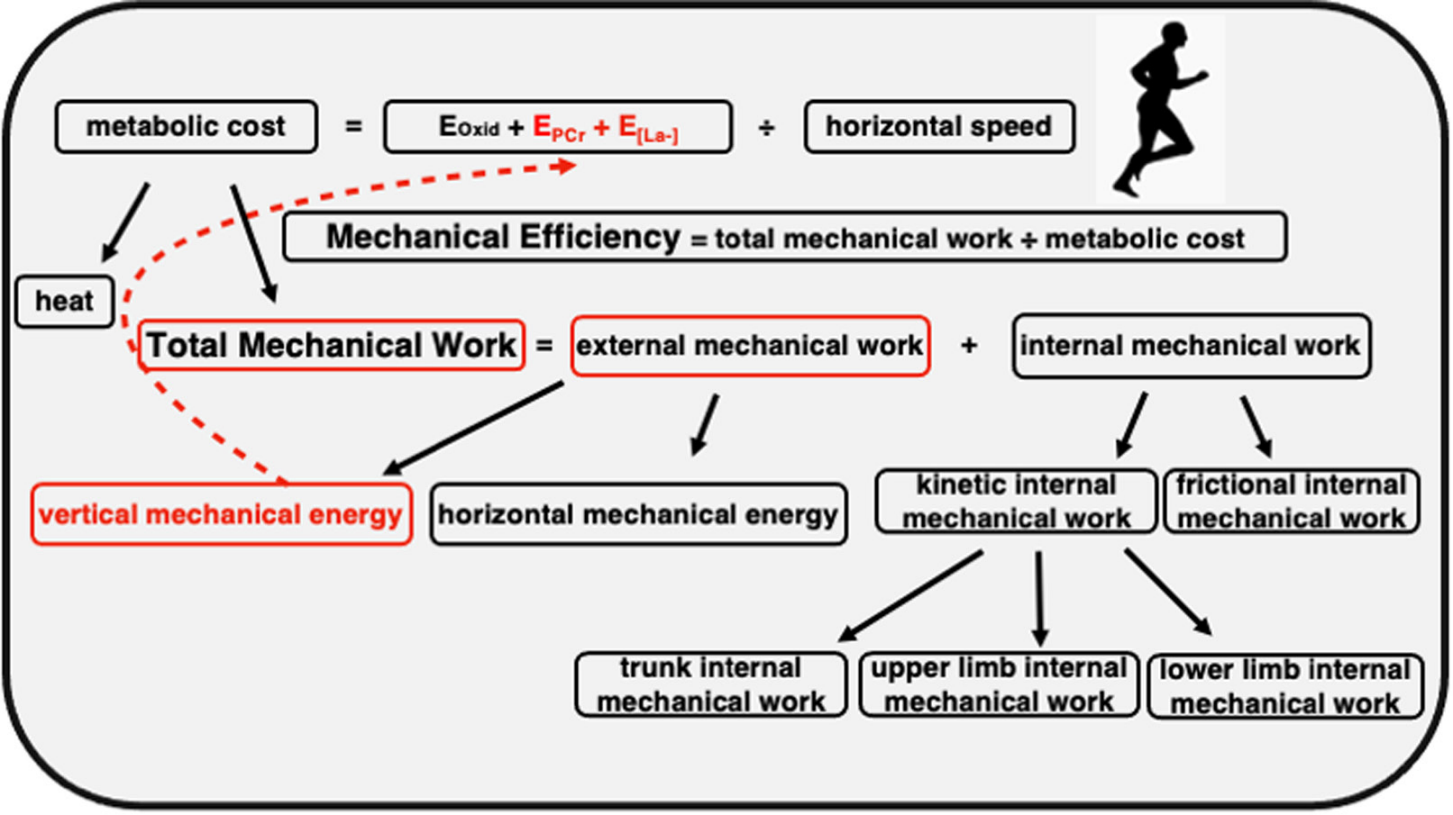
Conceptual model summarizing the findings. While no relationships were observed between physiologic, kinematic, and mechanical variables for the active group, the runners group show a correlation between vertical mechanical energy (influencing external mechanical work, and consequently, total mechanical work) and anaerobic pathways energy contribution. This result evidence the relevant influence of mechanical and kinematic variables on anaerobic energy expenditure (highlighted in red).

Finally, in Figure 2, a conceptual model summarizes our main results, showing a possible relation between mechanical and metabolic variables.

## 4 DISCUSSION

This study aimed to verify whether mechanical variables influence the anaerobic capacity outcome in running and whether these likely influences are dependent according to running experience. The main findings of this study were that the runners group showed a TTE approximately 19% lower, higher anaerobic capacity (16.6%) and stride length (21.4%), and lower contact phase duration (−11.3%) and vertical work (−29.9%), when compared with non‐runners. Our hypothesis was partly confirmed, in that our data showed that the vertical external work and contact time were lower in trained runners than non‐runners at high‐intensity effort. Furthermore, the anaerobic capacity was larger in trained runners. Nevertheless, in general, the running mechanics (total, external, and internal work) remain unchanged between trained runners versus non‐runners athletes. Notably, the vertical mechanical work related to anaerobic capacity meant that trained runners expend lesser energy to oscillate vertically the center of mass (generating less total, external, and vertical works) resulting in higher levels of absolute performance (i.e., speed) and anaerobic capacity, thus improving the transmission efficiency (Peyré‐Tartaruga & Coertjens, 2018). The conceptual model in Figure 2 summarizes the findings of this study.

The values of glycolytic and phosphagen pathways for active and runners groups corroborate with other studies that estimated the energy contribution of these metabolic pathways using the blood lactate concentration and an exponential fit on excess postexercise oxygen consumption curve, respectively (Zagatto, Miyagi, et al., 2017; Zagatto, Nakamura, et al., 2017). Considering that, the difference between anaerobic energy contributions values for both groups also corroborates with other studies in running (Zagatto, Miyagi, et al., 2017; Zagatto, Nakamura, et al., 2017), as well as in cycling (Dutra et al., 2020). It has been well evidenced on literature that anaerobic capacity is dependent of physical fitness and training status (Dutra et al., 2020; Zagatto, Miyagi, et al., 2017; Zagatto, Nakamura, et al., 2017) and that the time to exercise failure seems to be an inverse relationship (Dutra et al., 2020; Milioni et al., 2019; Zagatto, Miyagi, et al., 2017; Zagatto, Nakamura, et al., 2017). Likewise, our findings reported that the runners' group had a greater anaerobic capacity, but they have a lower time to exercise failure for the same exercise intensity (i.e., 115% of maximal). Similar results were reported by Zagatto, Miyagi, et al. (2017) and Zagatto, Nakamura, et al. (2017) when compared the anaerobic capacity and TTE at 115% of the intensity associated with V̇O_2max_, with endurance runners showing a running performance of 119.2, 154.8 s for the moderate‐trained group, 163.3 s for active groups, and 176.2 s for rugby professional players, while the rugby players had a higher anaerobic capacity compared to other groups. Dutra et al. (2020) also reported a higher anaerobic capacity measured on cycling for trained cyclists compared recreationally trained and finally untrained subjects. Beneke et al. (2011) have reported that during exercise intensity at and above of the maximal lactate steady state, international endurance trained athlete has a higher glycolytic rate (i.e., mmol·min^−1^) and a carbohydrate demand of approximately 75% higher than not specifically trained subjects. In this study, despite to perform the effort in the same intensity relative to vV̇O_2max_, the absolute intensity (e.g., running velocity or cycling power) were very different (i.e., exercise intensity at 115% of vV̇O_2max_ corresponded to 20.4 ± 1.4 km·h^−1^ for runners and 16.5 ± 1.3 km·h^−1^ for active subjects). Therefore, considering that the subjects with higher physical fitness have higher anaerobic capacity and other physiologic adaptations to produce and use energy during a maximal effort (higher enzymatic activity, glycolytic rate, carbohydrate demand, and others) as well as produce more metabolic wastes that are associated to effort failure (i.e., hydrogens ions, phosphate inorganic, and other) (Hureau et al., 2022), could explain the lower TTE for runners. In the same way, the effort movement pattern such as the running biomechanical, seems to alter the pattern of force, and thus alter the pattern of force, external and internal power outputs, metabolic responses such oxygen uptake demand and lactate production, decreasing the time to effort failure performance. Thus, it is likely effect over anaerobic capacity according to training status and running biomechanical could be established.

The lack of significant correlation (i.e., Pearson correlation and stepwise multiple regression) between anaerobic capacity and the kinematic and mechanical variables are in agreement with the findings by Andrade et al. (2015) and Minahan et al. (2007) and which did not find correlations between the anaerobic capacity estimated by maximal accumulated oxygen deficit and mechanical power calculated during Wingate test and running anaerobic sprint test, mainly due to oxidative contribution during the effort. In fact, despite the kinematic and mechanical variables are associated to performance (da Rosa, Oliveira, et al., 2019; Folland et al., 2017; Tartaruga et al., 2012), which was also observed in this study, no relevant impact was detected over anaerobic capacity.

Only the vertical work was into the regression for trained runners. It is generally believed that with lower vertical work varying, lesser energy would be dissipated for movement in this axis and directly for movement on horizontal axis, likely improving the lactate production and oxygen consumption uptake. Indeed, vertical external work appears to be a mechanical determinant of running (Cavagna et al., 1997, 2008). At submaximal running intensities, vertical external work is increased in trained runners. Interestingly, at high (supramaximal) speeds, this relationship is reversed. Thus, trained runners with higher performance show a specific technical adaptation to minimize the production of vertical work and, in turn, to potentiate the task of directing the body forward. Conversely, non‐runners were not able to make use of this strategy. Similar results were found in terms of force production at initial phase of sprint in athletes (Samozino et al., 2016). Future studies should explore whether this pattern of mechanical work generation remains at maximal speed phase of sprint. This study provides support for the mechanical effectiveness hypothesis (lower vertical work associated to invariant horizontal work) as a mechanical determinant of performance after the initial phase of sprint, at the highest speed of sprint. The invariance of horizontal external work corroborates previous findings related to aging (Cavagna et al., 2008) and altered stride frequency (Cavagna et al., 1997). Similar anaerobic capacity also confirms that runners can achieve better performances with better transmission efficiency (Peyré‐Tartaruga & Coertjens, 2018). Thus, metabolic (mainly anaerobic) energy is more efficiently used for running at supramaximal intensities through lower vertical work generation by controlling vertical body sway during the step (Figure 2).

## 5 CONCLUSION

In conclusion, the kinematic variables are not correlated with anaerobic capacity for untrained subjects and no predictive model was fitted, but for trained, there are negative correlation with external power, and total, external, vertical, and horizontal works and the only vertical work and phosphagen energy parameters could be used to prediction of anaerobic capacity. In this way, despite of results in different outcomes between training status, the anaerobic capacity is not affected by kinematics and mechanical parameters for active person, and for trained runner, only the vertical work had a relevant impact.

## AUTHOR CONTRIBUTIONS

Alessandro Moura Zagatto—conception or design of the work. Joel Abraham Martínez González performed the acquisition of data for the work. Alessandro Moura Zagatto, Joel Abraham Martínez González, Rodrigo Araujo Bonetti de Poli, Leonardo de los Santos Bloedow, and Leonardo Peyré‐Tartaruga performed the analysis and interpretation of data for the work. Alessandro Moura Zagatto and Leonardo Peyré‐Tartaruga wrote this manuscript and Fabio Augusto Barbieri e Rodrigo Araujo Bonetti de Poli revised critically for important intellectual content. All authors read and approved the final manuscript.

## FUNDING INFORMATION

This work was supported by the National Council for Scientific and Technological Development (CNPq)—Brazil, [Grant No. 422193/2016‐0] and Sao Paulo Research Foundation (FAPESP) Process number 13/12940‐8 and 19/17445‐1.

## CONFLICT OF INTEREST

Zagatto AM, González JAM, de Poli RAB, Barbieri FA, Bloedow LS, and Tartaruga LP declare that they have no conflicts of interest relevant to the content of this study.

## ETHICS APPROVAL

All procedures were approved by Sao Paulo State University Research Ethics Board (protocol no. 1.846.716) and were conducted according to the Declaration of Helsinki.
